# Docetaxel and gemcitabine activity in NSCLC cell lines and in primary cultures from human lung cancer

**DOI:** 10.1038/sj.bjc.6690737

**Published:** 1999-10

**Authors:** W Zoli, L Ricotti, M Dal Susino, F Barzanti, G L Frassineti, S Folli, A Tesei, F Bacci, D Amadori

**Affiliations:** Divisione di Oncologia Medica, Servizio di Anatomia-Istologia-Patologia, Ospedale ‘GB Morgagni-L Pierantoni’, viale Forlanini 34, Forlì, 47100, Italia; Istituto Oncologico Romagnolo Forli, Largo de Calboli 14, Forlì, 47100, Italia; Divisione di Chirurgia Toracica, Ospedale ‘GB Morgagni-L. Pierantoni’, piazzale Solieri 1, Forlì, 47100, Italia

**Keywords:** combination regimens, docetaxel, gemcitabine, NSCLC cell lines, NSCLC primary cultures, preclinical therapy

## Abstract

The activity of the following drugs was investigated in two established NSCLC cell lines: docetaxel, gemcitabine, vinorelbine, paclitaxel, doxorubicin (0.01, 0.1, 1 μg ml^−1^), cisplatin, ifosfamide (1, 2, 3 μg ml^−1^) and carboplatin (2, 4, 6 μg ml^−1^). The cytotoxic activity was evaluated by the sulphorhodamine B assay. The two most active drugs, docetaxel and gemcitabine, used singly and in association, were investigated as a function of treatment schedule. The sequence docetaxel→gemcitabine produced only a weak synergistic interaction in RAL but a strong synergism in CAEP cells. The synergistic interaction increased in both cell lines after a 48-h washout between the drug administrations. Flow cytometric analysis showed that in docetaxel→gemcitabine sequence, docetaxel produced a block in G2/M phase and, after 48 h, provided gemcitabine with a large fraction of recovered synchronized cells in the G1/S boundary, which is the specific target phase for gemcitabine. Conversely, simultaneous treatment induced an antagonistic effect in both cell lines, and the sequential scheme gemcitabine→docetaxel produced a weak synergistic effect only in RAL cells. Moreover, the synergistic interaction disappeared when washout periods of 24 or 48 h between two drug administrations were adopted. The synergistic activity of docetaxel→ 48-h washout→gemcitabine was confirmed in 11 of 14 primary cultures, which represents an important means of validating experimental results before translating them into clinical practice. © 1999 Cancer Research Campaign


British Journal of Cancer (1999) 81(4), 609–615
© 1999 Cancer Research Campaign
Article no. bjoc.1999.0737

### Docetaxel and gemcitabine activity in NSCLC cell lines and in primary cultures from human lung cancer

**W Zoli[1], L Ricotti[3], M Dal Susino[3], F Barzanti[3], GL Frassineti[1], S Folli[4], A Tesei[3], F Bacci[2] and D Amadori[1]**

[1]Divisione di Oncologia Medica, e [2]Servizio di Anatomia-Istologia-Patologia, Ospedale 'GB Morgagni-L Pierantoni', viale Forlanini 34, 47100 Forlì, Italia; [3]Istituto Oncologico Romagnolo Forli, Largo de Calboli 14, 47100 Forlì, Italia; [4]Divisione di Chirurgia Toracica, Ospedale 'GB Morgagni-L. Pierantoni', piazzale Solieri 1, 47100 Forlì, Italia

**Summary** The activity of the following drugs was investigated in two established NSCLC cell lines: docetaxel, gemcitabine, vinorelbine, paclitaxel, doxorubicin (0.01, 0.1, 1 μg ml$^{-1}$), cisplatin, ifosfamide (1, 2, 3 μg ml$^{-1}$) and carboplatin (2, 4, 6 μg ml$^{-1}$). The cytotoxic activity was evaluated by the sulphorhodamine B assay. The two most active drugs, docetaxel and gemcitabine, used singly and in association, were investigated as a function of treatment schedule. The sequence docetaxel→gemcitabine produced only a weak synergistic interaction in RAL but a strong synergism in CAEP cells. The synergistic interaction increased in both cell lines after a 48-h washout between the drug administrations. Flow cytometric analysis showed that in docetaxel→gemcitabine sequence, docetaxel produced a block in G2/M phase and, after 48 h, provided gemcitabine with a large fraction of recovered synchronized cells in the G1/S boundary, which is the specific target phase for gemcitabine. Conversely, simultaneous treatment induced an antagonistic effect in both cell lines, and the sequential scheme gemcitabine→docetaxel produced a weak synergistic effect only in RAL cells. Moreover, the synergistic interaction disappeared when washout periods of 24 or 48 h between two drug administrations were adopted. The synergistic activity of docetaxel→ 48-h washout→gemcitabine was confirmed in 11 of 14 primary cultures, which represents an important means of validating experimental results before translating them into clinical practice. © 1999 Cancer Research Campaign

**Keywords:** combination regimens; docetaxel; gemcitabine; NSCLC cell lines; NSCLC primary cultures; preclinical therapy

Non-small-cell lung cancer (NSCLC) is considered one of the most chemoresistant tumours, and in a recent overview the pessimism about the absolute survival benefits from chemotherapy was underlined (Non-small Cell Lung Cancer Collaborative Group, 1995). In particular, the meta-analysis revealed a survival benefit of 10% at 1 year in a supportive care setting and an increased median survival of 1.5 months in patients treated with platinum-based chemotherapy regimens, thus emphasizing the need for new, effective drugs and drug combination regimens.

Phase I–II clinical studies have shown that new drugs such as gemcitabine and taxanes, used singly (Carino et al, 1997; Cortes-Funes et al, 1997; Belani et al, 1998; Boyer et al, 1998; Natale et al, 1998; Takada et al, 1998) or in combination (Georgoulias et al, 1997*a*, 1997*b*), are active in NSCLC. Clinical protocols for cancer chemotherapy tend to use two or more drugs rather than single agents. Polychemotherapeutic protocol design is very complex. It is mainly based on information derived from experimental in vitro and in vivo studies and has favoured combinations of drugs with complementary mechanisms of action. Conversely, drug delivery schedules are planned without experimental preclinical information.

Preclinical studies have shown different interaction patterns of cisplatin and gemcitabine (Peters et al, 1995; van Moorsel et al, 1998), and paclitaxel and gemcitabine (Kroep et al, 1998) activity as a function of treatment schedule in some human tumour cell lines. Moreover, recent studies have shown the importance of recognizing specific perturbations induced by the different drugs on cell cycle when designing combination or sequential therapies in order to increase additive or synergistic effects and avoid antagonistic effects (Hahn et al, 1993; Theodossiou et al, 1998; Zoli et al, 1999).

We investigated the cytotoxic activity of docetaxel and gemcitabine, as well as their interaction as a function of treatment schedule and attributed their activity to induced cell cycle perturbations. With a view to translating preclinical information to clinical practice, the study was conducted on two cell lines derived from an epidermoid carcinoma and from an adenocarcinoma obtained and characterized in our laboratory, and on primary lung cancer cultures, considered the in vitro system which best reproduces the biology of clinical tumours.

*Received 5 August 1998*
*Revised 7 April 1999*
*Accepted 9 April 1999*

*Correspondence to:* W Zoli

### MATERIALS AND METHODS

### Established cell lines

The study was performed in two established NSCLC cell lines (obtained and characterized in our laboratory) representative of different lung cancer histotypes: the CAEP cell line derived from an epidermoid carcinoma and the RAL cell line derived from an adenocarcinoma (Gasperi-Campani et al, 1998). Cells were maintained as a monolayer at 37°C and subcultured weekly. Culture medium was composed of Dulbecco's modified Eagle's medium (DMEM)/Ham's F12 (1:1) supplemented with fetal calf serum (FCS; 10%), glutamine (2 mM), non-essential amino acids (1%) and insulin (10 μg ml$^{-1}$). Cells in the exponential growth phase were used for all the experiments.

### Primary cell cultures

Tumour material was obtained from 14 patients who underwent thoracotomy for primary lung cancer: six adenocarcinomas, four squamous cell carcinomas, three atypical and one typical carcinoids. The areas of gross necrosis were removed from samples, and tumour tissue was carefully minced in a small volume of culture medium (Ham's F12 supplemented with FCS (12%), L-glutamine (1%), insulin (1%), polymyxin B (50 U $ml^{-1}$), fungizone (5 μg $ml^{-1}$) and penicillin–streptomycin (50 U $ml^{-1}$) and reduced to fragments of about 1 mm. Samples were repeatedly passed through hypodermic needles of decreasing diameter. The resulting suspension was filtered through a nylon mesh (50 gauge), and a suspension consisting of single cells or small groups of cells was obtained. Cells were then washed two or three times in culture medium, collected by centrifugation for 5 min at 200 ***g*** and finally resuspended in fresh medium [DCCM supplemented with FCS (0.5%), 3,3′-triiodo-L-thyronine ($1 \times 10^{-8}$ M), epidermal growth factor (5 ng $ml^{-1}$), insulin (1%), glutamine (1%), hydrocortisone (0.1 μg $ml^{-1}$), 17β-estradiol ($1 \times 10^{-9}$ M), choleric toxin (1 ng $ml^{-1}$)] to perform the sulphorhodamine B (SRB) assay.

### Drugs

Vinorelbine (Pierre Fabre Pharma, Milan, Italy), carboplatin (Bristol Meyers Squibb, Latina, Italy), and docetaxel (Rhone-Poulenc Rorer, Varese, Italy) were diluted with sterile physiological solution at a concentration of 10 mg $ml^{-1}$, paclitaxel (Bristol Meyers Squibb) at 6 mg $ml^{-1}$, gemcitabine (Lilly, Sesto Fiorentino [FI], Italy, doxorubicin (Pharmacia, Milan, Italy) and ifosfamide (Asta Medica, Milan, Italy) at 1 mg $ml^{-1}$, and cisplatin (Iketon, Milan, Italy) at a concentration of 0.5 mg $ml^{-1}$, divided into aliquots, and stored at –20°C. Drug stocks were freshly diluted in culture medium before any experiment.

### In vitro chemosensitivity assay

The SRB assay according to the method of Skehan et al (1990) was used. Briefly, cells in the exponential phase of growth were collected by trypsinization, counted and plated in 96-well flat-bottomed microtitre plates (100 μl of cell suspension per well). Experiments were run in octoplet, and each experiment was repeated three times. Eighteen to 24 h after plating (a sufficient time for exponential growth recovery), 100 μl of culture medium containing or not the specific drugs were added to the wells. At the end of drug exposure, cells were fixed with 50% trichloroacetic acid at 4°C (10 μl per well, final concentration 10%) for 1 h. After five washes with tap water, cells were stained with 0.4% SRB dissolved in 1% acetic acid (50 μl per well) for 30 min and subsequently washed four times with 1% acetic acid to remove unbound stain. Plates were air-dried, and bound protein stain was solubilized with 100 μl of 10 mM unbuffered Tris base [Tris (hydroxymethyl)aminomethane]. The optical density of treated cells was read, at a wavelength of 540 or 510 nm, by means of a fluorescence plate reader.

### *Single drug exposure*

After preliminary experiments, drugs were used at scalar concentrations of 0.01, 0.1 and 1 μg $ml^{-1}$ for docetaxel and gemcitabine. Cells were exposed to the single drugs for 48 h.

### *Drug combinations*

Docetaxel and gemcitabine were tested using different combination and sequence schedules. Exposure time to each of the two drugs was 24 h. Docetaxel and gemcitabine were tested at all three concentrations (0.01, 0.1 and 1 μg $ml^{-1}$) in combination schemes and when they were used as the first drug in the sequential schemes. The lowest concentration (0.01 μg $ml^{-1}$) was used when docetaxel or gemcitabine was administered as the second drug in the sequential schemes.

Primary cell cultures were treated with the drug combination, schedule and timing that proved most effective in the established cell lines.

**Table 1** Cytotoxic effect of drugs on CAEP and RAL cell lines

| Drugs | Concentrations (μg $ml^{-1}$) | Mean $IC_{50}$ (μg $ml^{-1}$)[a] | |
|---|---|---|---|
| | | CAEP | RAL |
| Doxetaxel | 0.01, 0.1, 1 | 0.030 ± 0.01 | 0.085 ± 0.01 |
| Gemcitabine | 0.01, 0.1, 1 | 0.034 ± 0.009 | 0.530 ± 0.10 |
| Navelbine | 0.01, 0.1, 1 | 0.037 ± 0.01 | Not reached |
| Paclitaxel | 0.01, 0.1, 1 | 0.360 ± 0.12 | 0.833 ± 0.06 |
| Doxorubicin | 0.01, 0.1, 1 | Not reached | Not reached |
| Cisplatin | 1, 2, 3 | Not reached | Not reached |
| Ifosfamide | 1, 2, 3 | Not reached | Not reached |
| Carboplatin | 2, 4, 6 | Not reached | Not reached |

[a]After a 48-h treatment.

### Flow cytometric analysis

For the analysis of cell cycle perturbations, exponentially growing cells were trypsinized, rinsed and plated ($3 \times 10^5$ cells per dish) into 60-mm Petri dishes and incubated for 18–24 h at 37°C before drug exposure. Medium was aspirated from the plates, and 0.01 μg $ml^{-1}$ of docetaxel or gemcitabine was added to the exponentially growing cells. Control dishes were cultured using the same conditions, with comparable media changes. After a 24-h exposure to the drugs, cells were trypsinized, washed twice with phosphate-buffered saline and resuspended in 1 ml of 4,6-diamino-2-phenylindole (DAPI). For the determination of DNA content and S phase cell fraction in primary cultures, surgical samples were minced in 2 ml of DAPI for 3 min. Cells from cell lines and human tumours were then filtered through a disposable 40-μm filter assembly (RATCOM, Inc., Miami, FL, USA). Human lymphocytes were utilized as internal standard. For every sample, 30 000 cells were analysed by flow cytometry (RATCOM), and the data obtained were elaborated using Modfit (DNA Modeling System) software.

### Statistical analysis

To quantify deviations from additive effects induced by the sequential administration of two drugs, a statistical Student's *t*-test was employed (Drewinko et al, 1976). For a given drug dose, we determined a surviving fraction (Sf) of cells: SfA for the first drug used in the sequential schemes and SfB for the second. Following combined administration, we determined SfAB. Additivity held, resulting in SfAB = SfA × SfB, so that our estimate of deviation from additivity was the quantity SfAB – (SfA × SfB). The ratio of

© 1999 Cancer Research Campaign

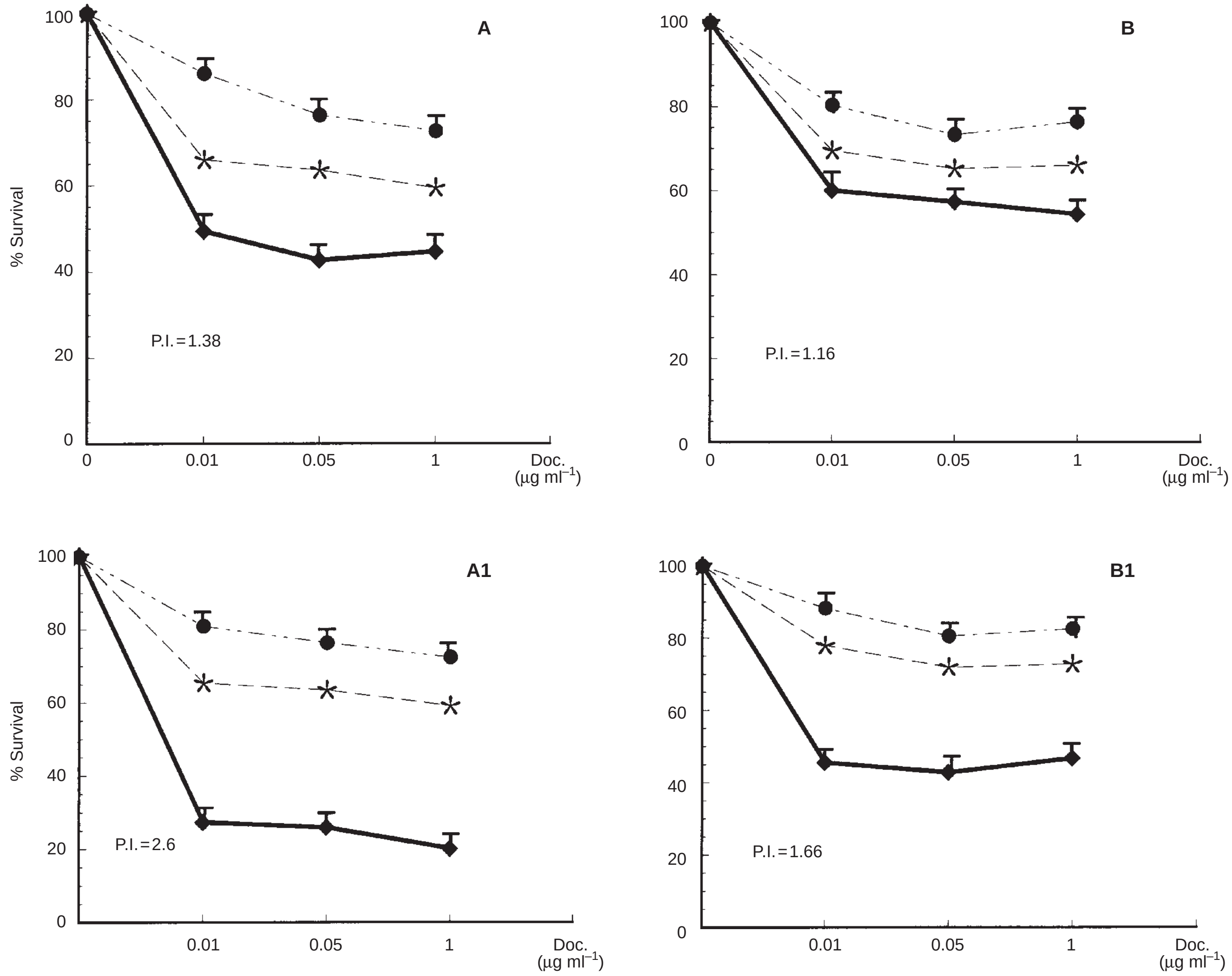

**Figure 1** Dose-response survival curves of CAEP (A and A1) and RAL (B and B1) cells exposed to: (A) docetaxel for 24 hr followed by gemcitabine (0.01 µg $ml^{-1}$) for 24 hr; (A1) docetaxel for 24 hr followed by a 48-hr washout and then gemcitabine (0.01 µg $ml^{-1}$) for 24 hr; (B) docetaxel for 24 hr followed by gemcitabine (0.01 mg ml-1) for 24 hr; (B1) docetaxel for 24 hr followed by a 48-hr washout and then gemcitabine (0.01 µg $ml^{-1}$) for 24 hr. Docetaxel, observed survival – •• – •• – ● – •• – •• –; docetaxel→gemcitabine, – – – – ⁎ – – – – expected survival, —— ♦ ——observed survival.

differences between observed versus expected survival and the square root of the relative variances for all drug combinations examined were, in fact, distributed normally, with the average equalling 0 and the variance equalling 1. The results obtained were defined according to the following criteria: SfAB = SfA × SfB indicated an additive effect, SfAB < SfA × SfB, a synergistic effect, and SfAB > SfA × SfB, an antagonistic effect. In drug combination studies, the performance index (PI) statistic model was used to evaluate type of interaction (Drewinko et al, 1976).

### RESULTS

### Established cell lines

The CAEP cell line was generally more sensitive to all the drugs than the RAL cell line. In particular, of the eight tested drugs, docetaxel and gemcitabine were the two most effective in both cell lines (Table 1).

The 24-h treatment with docetaxel followed by 24-h with gemcitabine (Figure 1) produced only a weak synergistic interaction in the RAL cell line (PI = 1.16) but a strong synergism in the CAEP cell line (PI = 1.38). The synergistic interaction following the sequence was further increased by a 48-h washout in between the two-drug treatments in RAL cells (PI = 1.65) and even more so in the most sensitive cell line, CAEP (PI = 2.6) (Figure 1).

The sequential scheme of a 24-h treatment with gemcitabine immediately followed by docetaxel produced (Figure 2) a weak synergistic effect only in the RAL cell line (PI = 1.15). The synergistic interaction disappeared when washout periods of 24 or 48 h in between the two-drug treatments were adopted (Figure 2).

The simultaneous administration of docetaxel and gemcitabine induced (Figure 3) an antagonistic interaction in both cell lines at all docetaxel concentrations. The antagonistic effect was consistently observed when a single concentration of docetaxel and increasing concentrations of gemcitabine were tested (data not shown).

Cell cycle perturbations were analysed by flow cytometric analysis in an attempt to explain the mechanism underlying the synergistic interaction. In RAL cells, a 24-h treatment with gemcitabine caused an increase in G0/G1 phase cells and a dramatic decrease in the G2/M phase, which were still present at the 72-h

© 1999 Cancer Research Campaign

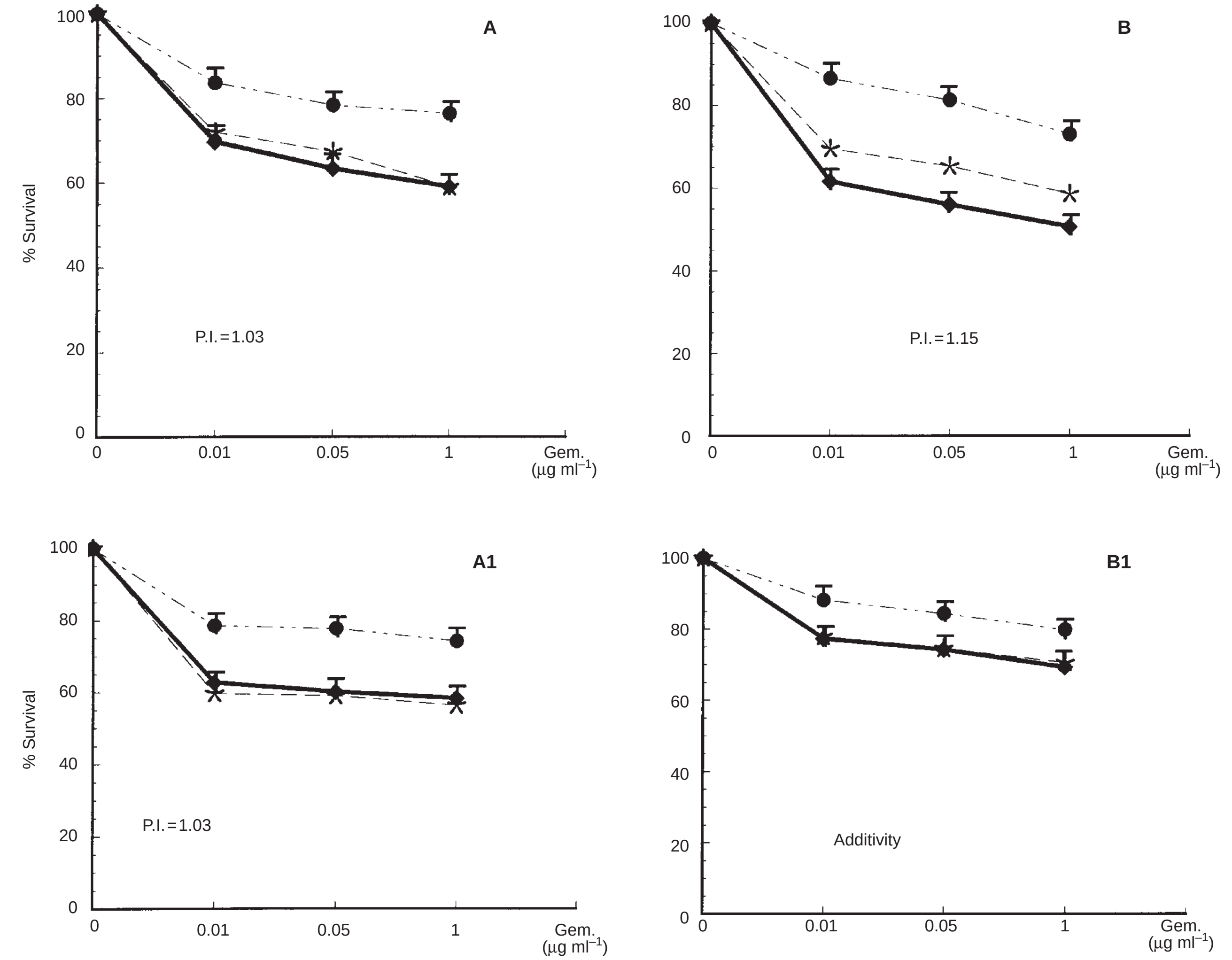

**Figure 2** Dose-response survival curves of CAEP (A and A1) and RAL (B and B1) cells exposed to: (A) gemcitabine for 24 hr followed by docetaxel (0.01 µg ml−1) for 24 hr; (A1) gemcitabine for 24 hr followed by a 48-hr washout and then docetaxel (0.01 µg ml−1) for 24 hr; (B) gemcitabine for 24 hr followed by docetaxel (0.01 µg ml−1) for 24 hr; (B1) gemcitabine for 24 hr followed by a 48-hr washout and then docetaxel (0.01 µg ml−1) for 24 hr. Gemcitabine, observed survival – •• – •• – ● – •• – •• –; docetaxel→gemcitabine, –––– * –––– expected survival, —— ♦ ——observed survival.

washout. The S phase cell fraction decreased slightly but had completely recovered after the 72-h washout.

In the CAEP cell line, an increase in the G0/G1 cell fraction was also observed in concomitance with a progressive reduction in G2/M cells, for up to 48 h, and a partial recovery starting from the 72-h washout. The S phase cell fraction was not affected (Table 2). Docetaxel induced a characteristic cell block in the G2/M phase after a 24-h treatment (more evident in CAEP than in RAL cells), which increased after a 24-h washout and progressively recovered within 72 h at the pre-wash levels (Table 3).

### Primary cell cultures

The antiproliferative effect of the most effective sequential treatment, docetaxel → 48-h washout → gemcitabine, was tested in 14 primary cell cultures obtained from surgical material of untreated lung cancer patients. Results (Table 4) showed a synergistic effect in 11 cancers (80%), an additive effect in two and an antagonistic effect in one case. The additive interactions were observed in a squamous carcinoma and in a typical carcinoid lesion. The only antagonistic effect was seen in an adenocarcinoma. In this limited case series, the type of interaction did not appear to be related to FCM–S phase cell fraction or DNA content.

### DISCUSSION

Vinblastine, ifosfamide, cisplatin, vindesine and mitomycin, which are among the most active conventional cytotoxic agents used in monochemotherapy to treat NSCLC, induce objective tumour response rates of about 15% in patients. It has been seen that the association of cisplatin with one of the other drugs only slightly improves survival at 5 years (Non-small Cell Lung Cancer Collaborative Group, 1995). Recently, new compounds have been proposed which have raised some hopes for NSCLC patient treatment. Among the most effective of these are the topoisomerase I poisons, topotecan and irinotecan (which are both camptothecin derivatives), the tubulin stabilizers such as paclitaxel and docetaxel, the new vinca alkaloid, vinorelbine and, finally, the antimetabolite, gemcitabine. When used as single agents, these new drugs have yielded response rates of more than 20% (Feigal et al, 1993; Le Chevalier, 1996).

*© 1999 Cancer Research Campaign*

**Table 2** Percentage of cells in different cell cycle phases at different times after a 24-h treatment with gemcitabine (0.01 µg ml$^{-1}$)

| Cell cycle phase | Control samples | Times following treatment | | | |
|---|---|---|---|---|---|
| | | 0 h | 24 h | 48 h | 72 h |
| *RAL* | | | | | |
| $G_0$-$G_1$ | 50.2 | 70.1 | 70.2 | 69.7 | 67.4 |
| S | 35.6 | 28.0 | 28.0 | 29.1 | 32.3 |
| $G_2$-M | 14.2 | 1.9 | 1.8 | 1.2 | 0.3 |
| Debris | 9.6 | 23.7 | 5.3 | 5.7 | 8.6 |
| *CAEP* | | | | | |
| $G_0$-$G_1$ | 55.1 | 65.4 | 63.6 | 70.7 | 63.5 |
| S | 26.1 | 24.5 | 26.5 | 28.1 | 30.5 |
| $G_2$-M | 18.8 | 10.1 | 9.9 | 1.2 | 6.0 |
| Debris | 21.6 | 22.4 | 12.2 | 5.7 | 6.4 |

**Table 3** Percentage of cells in different cell cycle phases at different times after a 24-h treatment with docetaxel (0.01 µg ml$^{-1}$)

| Cell cycle phase | Control samples | Times following treatment | | | |
|---|---|---|---|---|---|
| | | 0 h | 24 h | 48 h | 72 h |
| *RAL* | | | | | |
| $G_0$-$G_1$ | 50.2 | 30.1 | 11.4 | 13.4 | 20.2 |
| S | 35.5 | 34.4 | 22.2 | 28.4 | 42.6 |
| $G_2$-M | 14.3 | 35.5 | 66.4 | 58.2 | 37.2 |
| Debris | 10.0 | 17.4 | 58.8 | 64.1 | 57.2 |
| *CAEP* | | | | | |
| $G_0$-$G_1$ | 55.1 | 20.5 | 11.2 | 13.4 | 34.1 |
| S | 26.0 | 21.5 | 10.2 | 27.4 | 27.0 |
| $G_2$-M | 18.9 | 58.0 | 78.6 | 59.2 | 48.9 |
| Debris | 21.6 | 50.7 | 75.6 | 54.2 | 36.0 |

In previous studies on human breast cancer cell lines and primary breast cancer cultures, we showed (Amadori et al, 1996) that treatment with doxorubicin followed by paclitaxel was more cytotoxic than simultaneous drug administration or the inverse sequence of paclitaxel followed by doxorubicin. We also showed that the sequence defined at a preclinical level actually resulted in a high therapeutic efficacy in advanced breast cancer patients (Amadori et al, 1996; Frassineti et al, 1997). In a further preclinical study on human breast cancer cell lines (Zoli et al, 1999), we observed a major therapeutic improvement using the sequence doxorubicin → paclitaxel → 48-h washout → gemcitabine. The schedule-dependent activity of multidrug regimens observed by us and other authors thus emphasizes the importance of preclinical studies (Peters et al, 1995; Kroep et al, 1998; van Moorsel et al, 1998).

In the present study, we used two NSCLC cell lines which reproduce a clinical situation since they proved to be highly sensitive to docetaxel and gemcitabine, in agreement with results from preclinical and phase I–II studies on NSCLC (Carino et al, 1997; Cortes-Funes et al, 1997; Boyer, 1998; Natale et al, 1998; Takada et al, 1998). The sequence gemcitabine → 48-h washout → docetaxel produced a low synergistic effect in both cell lines. This can, in part, be attributed to a block induced by gemcitabine in the G0/G1 phase that recovered after 72 h. Such a block may prevent exposure of the cells to the cytotoxic effect of docetaxel when the drug is administered immediately or 48 h after gemcitabine treatment. Conversely, the cytotoxic effect obtained with the opposite schedule, docetaxel → 48-h washout → gemcitabine, produced an evident synergistic effect in CAEP and a strong synergistic effect in RAL cell lines. Cell cycle perturbation analysis following this treatment schedule indicated that docetaxel produced an initial block in the G2/M phase, thus providing a large fraction of recovered synchronized cells in the G1/S boundary, which is the specific target phase for the antimetabolite (Hertel et al, 1990; Theodossiou et al, 1998), for the subsequent treatment with gemcitabine. It should be pointed out that the synergistic effect was already present at the lowest drug concentration.

Our findings are in agreement with those of Theodossiou et al (1998), who observed an antagonistic effect of gemcitabine and paclitaxel when administered simultaneously in the A549 lung cancer cell line. The same study showed a slightly less than additive cytotoxic effect when gemcitabine administration preceded that of paclitaxel, or after the inverse sequence. Conversely, using

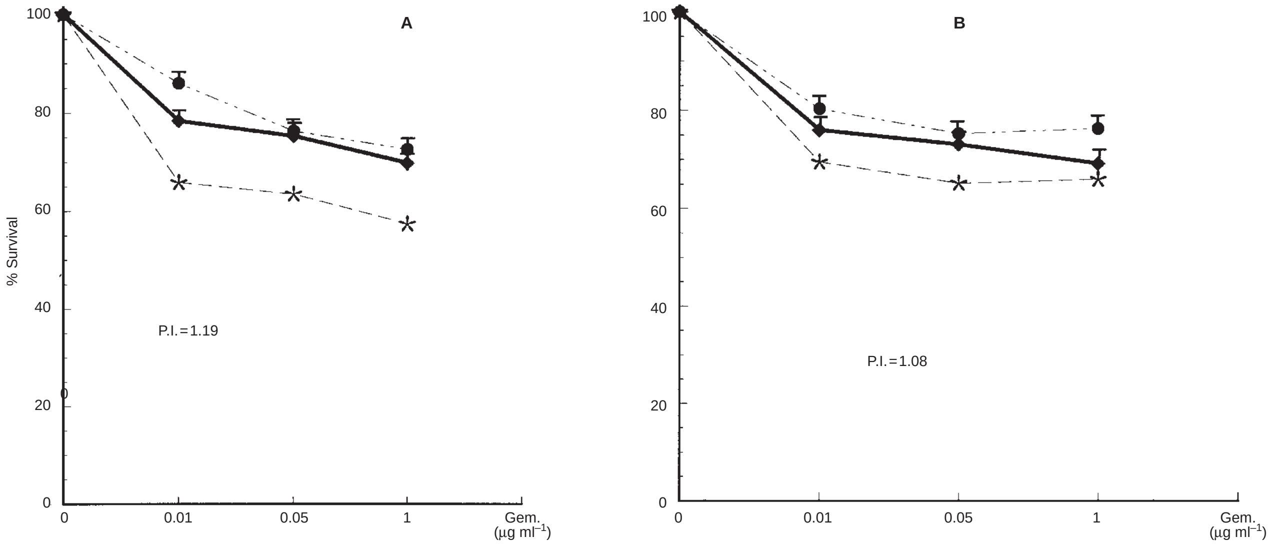

**Figure 3** Dose-response survival curves of CAEP (A) and RAL (B) cells exposed to: docetaxel plus gemcitabine (0.01 µg ml$^{-1}$) for 24 hr. Docetaxel, observed survival – •• – •• – ● – •• – •• –; docetaxel→gemcitabine, –––– ⋆ –––– expected survival, —— ♦ ——observed survival.

*© 1999 Cancer Research Campaign*

**Table 4** Cytotoxic effects of sequential treatment, docetaxel (0.01, 0.1 and 1 μg ml$^{-1}$ for 24 h) followed by a 48-h washout and then gemcitabine (0.01 μg ml$^{-1}$ for 24 h), observed in 14 primary lung cancer cultures

| Sample | Histotype | Cytotoxic effect | Performance index | DNA content | FCM-S (%) |
|---|---|---|---|---|---|
| 1 | Adenocarcinoma | Synergistic | 2.40 | Multiploid | NA[a] |
| 2 | Adenocarcinoma | Synergistic | 1.55 | Near-diploid | 9.7 |
| 3 | Adenocarcinoma | Synergistic | 1.46 | Hyperdiploid | 16.8 |
| 4 | Adenocarcinoma | Synergistic | 1.37 | Multiploid | 3.8 |
| 5 | Adenocarcinoma | Synergistic | 1.31 | Multiploid | NA |
| 6 | Adenocarcinoma | Antagonistic | 1.29 | Multiploid | NA |
| 7 | Squamous cell ca. | Synergistic | 4.03 | Near-diploid | 11.5 |
| 8 | Squamous cell ca. | Synergistic | 3.53 | Multiploid | NA |
| 9 | Squamous cell ca. | Synergistic | 1.41 | Near-diploid | 11.1 |
| 10 | Squamous cell ca. | Additive | – | Near-diploid | 11.4 |
| 11 | Atypical carcinoid | Synergistic | 4.10 | Multiploid | 12.5 |
| 12 | Atypical carcinoid | Synergistic | 1.20 | Hypodiploid | 4.4 |
| 13 | Typical carcinoid | Additive | – | Hypodiploid | 1.0 |
| 14 | Neuroendocrine with atypical carcinoid | Synergistic | 1.56 | Hyperdiploid | 14.8 |

[a]Not assessable for the partial overlapping of DNA histograms belonging to the different clones.

the other taxane, docetaxel, and following the sequential treatment docetaxel–gemcitabine, we observed a synergistic cytotoxic effect that increased significantly when the second drug was given after a 48-h washout. This finding was confirmed in most of the primary cultures from clinical human lung cancer tumour we used, which represents an important step in validating preclinical results before translating them into clinical practice (Villa et al, 1992).

The results from the present study reinforce the importance, previously evidenced for other drugs and tumour types (Citro et al, 1991; Savini et al, 1992; Dogliotti et al, 1996; De Lena et al, 1997; Silvestrini et al, 1997; Amadori et al, 1998; Fischel et al 1998; Van Moorsel et al 1998, 1999; Zoli et al, 1999) of preclinical research to define the best treatment scheduling. Considering the recently published results (Spiridonidis et al, 1998; Georgoulias et al, 1999), which show that gemcitabine and docetaxel can be safely used in combination, a protocol on advanced NSCLC will shortly be activated in Italy based on the findings from the present study.

### ACKNOWLEDGEMENTS

The authors thank Prof. Rosella Silvestrini for her invaluable scientific contribution and advice, and G Tierney for editing the manuscript. This work was supported by a grant from the Istituto Oncologico Romagnolo.

### REFERENCES

Amadori D, Frassineti GL, Zoli W, Milandri C, Tienghi A, Ravaioli A, Gentile A and Salzano E (1996) A phase I/II study of sequential doxorubicin and paclitaxel treatment of advanced breast cancer. *Semin Oncol* **23** (Suppl 11): S16–S22

Amadori D, Frassineti GL, De Matteis A, Mustacchi G, Santoro A, Cariello S, Ferrari M, Nascinben O, Nanni O, Lombardi A, Scarpi E and Zoli W (1998) Modulating effect of lonidamine on response to doxorubicin in metastatic breast cancer patients: results from a multicenter prospective randomized trial. *Breast Cancer Res Treat* **49**: 209–217

Belani CP (1998) Single agents in the second-line treatment of non-small cell lung cancer. *Semin Oncol* **25** (Suppl 8): S10–S14

Boyer MJ (1998) New chemotherapeutic agents in the treatment of non-small cell lung cancer: the Australian experience. *Chest* **113** (suppl 1): S24–S27

Crino L, Mosconi AM, Scagliotti G, De Marinis F, Darwish S, Calandri C, Adamo V, Scarcella L, Pucci F and Tonato M (1997) Salvage therapy with gemcitabine (GEM) in pretreated, advanced non-small cell lung cancer (NSCLC). *Proc Annu Meet Soc Clin Oncol* **16**: 1603

Citro G, Cucco C, Verdiana A and Zupi G (1991) Reversal of adriamycin resistance by lonidamine in a human breast cancer cell line. *Br J Cancer* **64**: 534–536

Cortes-Funes H, Martin C, Abrat R and Lund B (1997) Safety profile of gemcitabine, a novel anticancer agent, in non-small cell lung cancer. *Anticancer Drugs* **8**: 582–587

De Lena M, Lorusso V, Bottalico C, Brandi M, De Mitrio A, Catino A, Guida M, Latorre A, Leone B, Vallejo C and Gargano G (1997) Revertant and potentiating activity of lonidamine in patients with ovarian cancer previously treated with platinum. *J Clin Oncol* **15**: 3208–3213

Dogliotti L, Berruti A, Buniva T, Zola P, Bau MG, Farris A, Sarobba MG, Bottini A, Alquati P, Deltetto F, Gosso P, Monzeglio C, Moro G, Sussio M and Perroni D (1996) Lonidamine significantly increases the activity of epirubicin in patients with advanced breast cancer: results from a multicenter prospective randomized trial. *J Clin Oncol* **14**: 1165–1172

Drewinko B, Loo TL, Brown B, Gottlieb JA and Freireich EJ (1976) Combination chemotherapy in vitro with adriamycin. Observations of additive, antagonistic and synergistic effects when used in two-drug combinations on cultured human lymphoma cells. *Cancer Biochem Biophys* **1**: 187–195

Feigal EG, Christian M and Cheson B (1993) New chemotherapeutic agents in non-small cell lung cancer. *Semin Oncol* **20**: 185–201

Fischel JL, Etienne MC, Formento P and Milano G (1998) Search for the optimal schedule for the oxaliplatin/5-fluoruracil association modulated or not by folinic acid: preclinical data. *Clin Cancer Res* **4**: 2529–2535

Frassineti GL, Zoli W, Silvestro L, Serra P, Milandri C, Tienghi A, Gianni L, Gentile A, Salzano E and Amadori D (1997) Paclitaxel plus doxorubicin in breast cancer: an Italian expeirence. *Semin Oncol* **24**: (Suppl 17) S19–S25

Gasperi-Campani A, Roncuzzi L, Ricotti L, Lenzi L, Gruppioni R, Sensi A, Zini M, Zoli W and Amadori D (1998) Molecular and biological features of two new human cell lines from squamous and adenocarcinoma of the lung. *Cancer Gen Cytogen* **107**: 11–20

Georgoulias V, Kourousis C, Kakolyris S, Androulakis N, Dimopoulos MA, Papadakis E, Kotsakis T, Vardakis N, Kalbakis K, Meramveliotakis N and Hatzidaki D (1997*a*). Second-line treatment of advanced non-small cell lung cancer with paclitaxel and gemcitabine: a preliminary report on an active regimen. *Semin Oncol* **24** (Suppl 12): S61–S66

Georgoulias V, Kourousis C, Androulakis N, Kakolyris S, Dimopoulos MA, Bouros D, Papadimitriou C, Hatzakis K, Heras P, Kalbakis K, Kotsakis T, Vardakis N, Meramveliotakis N and Hatzidaki D (1997*b*). Docetaxel (Taxotere) and gemcitabine in the treatment of non-small cell lung cancer: preliminary results. *Semin Oncol* **24** (Suppl 14): S22–S25

Georgoulias V, Kourousis C, Androulakis N, Kakolyris S, Dimopoulos MA, Papadakis E, Bouros D, Apostolopaulou F, Papadimitriou C, Angelidou A, Hatzakis K, Kalbakis K, Kotsakis T, Vardakis N and Vlachonicolis J (1999) Front-line treatment of advanced non-small-cell lung cancer with docetaxel and gemcitabine: a multicenter phase II trial. *J Clin Oncol* **17**: 914–920

Hahn SM, Liebman JE, Cook J, Fisher J, Goldspiel B, Venzon D, Mitchell JB and Kaupfman D (1993) Taxol in combination with doxorubicin or etoposide. *Cancer* **72**: 2705–2711

 © 1999 Cancer Research Campaign

Hertel LW, Boder GB, Kroin JS, Rinzel SM, Poore GA, Todd GC and Grindey GB (1990) Evaluation of the antitumor activity of gemcitabine (2′,2′-difluoro-2′-deoxycytidine). *Cancer Res* **50**: 4417–4422

Le Chevalier T (1996) Single-agent activity of gemcitabine in advanced non-small cell lung cancer. *Semin Oncol* **23** (Suppl 10): 36–42

Natale RB (1998) Experience with new chemotherapeutic agents in non-small cell lung cancer. *Chest* **113** (Suppl 1): 32S–39S

Non-small Cell Lung Cancer Collaborative Group (1995) Chemotherapy in non-small cell lung cancer: a meta-analysis using updated data on individual patients from 52 randomised clinical trials. *Br Med J* **311**: 899–909

Peters GJ, Bergman AM, Ruiz van Haperen VWT, Veerman G, Kuiper CM and Braakhuis BJM (1995) Interaction between cisplatin and gemcitabine in vitro and in vivo. *Semin Oncol* **22** (Suppl 11): 72–79

Savini S, Zoli W, Nanni O, Volpi A, Frassineti GL, Magni E, Flamigni A, Amadori A and Amadori D (1992) In vitro potentiation by lonidamine of the cytotoxic effect of adriamycin on primary and established breast cancer cell lines. *Breast Cancer Res Treat* **24**: 27–34

Silvestrini R, Gornati D, Zaffaroni N, Bearzatto A and De Marco C (1997) Modulation by lonidamine on the combined activity of cisplatin and epidoxorubicin in human breast cancer cells. *Breast Cancer Res Treat* **42**: 103–112

Skehan P, Storeng R, Scudiero D, Monks A, McMahon J, Vistica D, Warren J, Bokesch H, Kenney S and Boyd MR (1990) New colorimetric cytotoxic assay for anticancer-drug screening. *J Natl Cancer Inst* **13**: 1107–1112

Spiridonidis CH, Laufman LR, Jones J, Rhodes VA, Wallace K and Nicol S (1998) Phase I study of docetaxel dose escalation in combination with fixed weekly gemcitabine in patients with advanced malignancies. *J Clin Oncol* **16**: 3866–3873

Takada M, Negoro S, Kudo S, Furuse K, Nishikawa H, Takada Y, Kamei T, Niitani H and Fukuoka M (1998) Activity of gemcitabine in non-small-cell lung cancer: results of the Japan gemcitabine group (A) phase II study. *Cancer Chemother Pharmacol* **41**: 217–222

Theodossiou C, Cook JA, Fisher J, Teague D, Liebmann JE, Russo A and Mitchell JB (1998) Interaction of gemcitabine with paclitaxel and cisplatin in human tumour cell lines. *Int J Oncol* **12**: 825–832

Van Moorsel CJA, Veerman G, Vermoken JB, Kroep JR, Van Groeningen CJ, Voorn DA, Catik A, Gall HE, Eeltink CM, Van der Vijgh WJF, Pinedo HM and Peters GJ (1998) Schedule dependent changes in the pharmacokinetics (PK) and pharmacodynamics of gemcitabine (GEM) and cisplatin (CP) in patients (pts) with solid tumors. *Proc Am Assoc Cancer Res* **39**: 188

Van Moorsel CJA, Pinedo HM, Veerman G, Guechev A, Smid K, Loves WJ, Vermoken JB, Postmun PE and Peters GJ (1999) Combination chemotherapy studies with gemcitabine and etoposide in non-small cell lung and ovarian cancer cell lines. *Biochem Pharmacol* 15; **57** (4): 407–415

Kroep JR, Voorn DA, Van Moorsel CJA, Giaccone G, Postmus PE, Rosing H, Beijnen JH, Gall H, Smit EF, Van Groeningen CJ, Pinedo HM and Peters GJ (1998) Pharmakinetics interaction between paclitaxel (TAX) and gemcitabine (GEM) in patients (pts) with non-small-cell lung cancer (NSCLC). *Proc Am Assoc Cancer Res* **39**: 188

Villa R, Zaffaroni N, Orlandi L, Costa A, Veronese S, Vaglini N and Silvestrini R (1992) Reliability of a primary system to test cytotoxic drug activity in human malignant melanoma. *Int J Oncol* **1**: 619–624

Zoli W, Ricotti L, Barzanti L, Dal Susino M, Frassineti GL, Milandri C, Casadei Giunchi D and Amadori D (1999) Schedule-dependent interaction of doxorubicin, paclitaxel and gemcitabine in human breast cancer cell lines. *Int J Cancer* **80**: 413–416

© 1999 Cancer Research Campaign 
